# All-Silicon Photoelectric Biosensor on Chip Based on Silicon Nitride Waveguide with Low Loss

**DOI:** 10.3390/nano13050914

**Published:** 2023-03-01

**Authors:** Yu Tang, Qian Luo, Yuxing Chen, Kaikai Xu

**Affiliations:** State Key Laboratory of Electronic Thin Films and Integrated Devices, University of Electronic Science and Technology of China, Chengdu 610054, China

**Keywords:** PN junction cascade light source, evanescent wave, silicon nitride waveguide, monolithic integrated

## Abstract

Compared to the widely used compound semiconductor photoelectric sensors, all-silicon photoelectric sensors have the advantage of easy mass production because they are compatible with the complementary metal-oxide-semiconductor (CMOS) fabrication technique. In this paper, we propose an all-silicon photoelectric biosensor with a simple process and that is integrated, miniature, and with low loss. This biosensor is based on monolithic integration technology, and its light source is a PN junction cascaded polysilicon nanostructure. The detection device utilizes a simple refractive index sensing method. According to our simulation, when the refractive index of the detected material is more than 1.52, evanescent wave intensity decreases with the growth of the refractive index. Thus, refractive index sensing can be achieved. Moreover, it was also shown that, compared to a slab waveguide, the embedded waveguide designed in this paper has a lower loss. With these features, our all-silicon photoelectric biosensor (ASPB) demonstrates its potential in the application of handheld biosensors.

## 1. Introduction

Optical sensors [1] and carrier-based sensors [2,3] have been studied extensively as media for humans to perceive things around them. Since the 1980s, scientists have explored light-based biosensors as a medium for human perception [4]. As one of the major branches of biosensors, photoelectric biosensors are essential for many application areas, such as medical diagnosis, agricultural ecology, environmental security, etc. [5,6,7,8]. They are used to detect various parameters, such as water and sun radiation. They have different sensing mechanisms, which are a refractive index, absorption, and fluorescence sensing [9,10,11]. A photoelectric biosensor can detect light quantity variation, such as the substance’s type, composition, and concentration. The photoelectric biosensor has a high detection sensitivity, simple operation, and low loss, and can be applied to real-time detection and monitor the dynamic process of biological reactions. However, at the moment, the photoelectric biosensors’ mobility, complexity, accuracy, and response time are insufficient to satisfy high-demand events accurately.

Therefore, researchers are committed to finding optoelectronic integrated biosensors with low loss, a fast response speed, miniature size, simple repeatability, and low cost [12,13,14,15,16,17,18]. In 2015, Chaudhuri et al. [19] conducted in-depth research regarding sensor miniaturization and designed a chip-sensing platform with heterogeneous integration on a silicon substrate. In 2017, Okhai et al. [14] studied biodetection waveguide technology and realized the micro-electro-optical biosensor on a chip. The advent of on-chip technology has brought small sizes and high integration. Due to extensive research, the performance of the light source [20], optical waveguide (OW) [21,22], and photodetector [23,24,25,26,27] of the on-chip microsystem have been significantly improved now.

Most light sources nowadays use III-V compound semiconductor light sources, such as gallium arsenide (GaAs) and indium phosphide (InP), which have high luminous efficiency. Nevertheless, they are not monolithic, and their growing process is complicated, which makes them expensive. On the other hand, silicon has dominated the semiconductor industry for years because its manufacturing process is mature and cost-effective. Thus, if silicon can be used to form the light sources, the cost of the devices will become much lower.

Si_3_N_4_ can be used to form the OW in a silicon-based optical system. In the visible band, Si_3_N_4_ is a medium refractive index material and compatible with the CMOS fabrication process. In addition, Si_3_N_4_ has a good chemical inertia [28], thermal stability, and low loss [29]. This makes Si_3_N_4_ a good candidate to serve as the OW in an on-chip sensor. In this paper, we developed an ASPB with monolithic integration technology, a simple process, miniature size, and low loss.

## 2. System Design and Device Structure 

**Figure 1:**
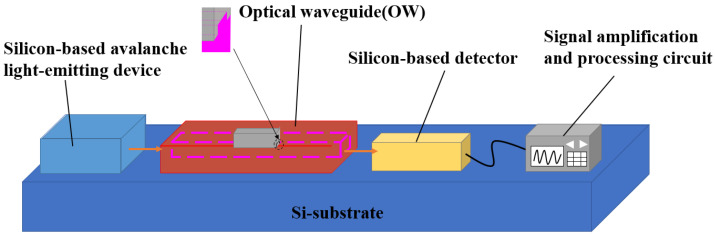
In ASPB, the light generated by the silicon-based avalanche light-emitting device was transmitted to the OW and coupled with the material to be detected, resulting in light attenuation. Then, the attenuated light was detected by the silicon-based detector and converted into an electrical signal. Finally, the sensing results were output by the processing circuit.

Figure 1 illustrates the sketch of the developed ASPB, which included silicon-based avalanche light-emitting devices, OWs, silicon-based detectors, signal amplification, and processing circuits.

**Figure 2:**
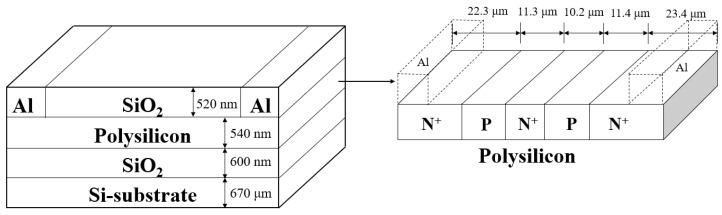
The structure of the polysilicon light source.

The luminous efficiency of the light source plays a decisive role in the performance of the whole ASPB. The structure of the PN junction cascade light source adopted in this paper is illustrated in Figure 2. The polysilicon light source comprised a monocrystalline silicon substrate layer, a silicon dioxide insulation layer, a polysilicon layer, and a silicon dioxide layer (from bottom to top). A 670 μm thick monocrystalline silicon layer was used as the substrate, and a 600 nm thick silicon dioxide layer was placed above it to isolate and support the monocrystalline substrate from the polysilicon layers. The polycrystalline silicon layer deposited on the silicon dioxide insulation layer had a thickness of 540 nm and served as the emitting region of the light source. Finally, the silicon dioxide layer deposited on the polysilicon had a thickness of 520 nm, preventing the polysilicon from oxidizing. The topmost silicon dioxide layer was etched at both ends, and metallic aluminum was deposited as the electrode. The polycrystalline silicon region had a 22.3 μm N^+^ region, an 11.3 μm P region, a 10.2 μm N^+^ region, an 11.4 μm P region, and a 23.4 μm N^+^ region (from left to right). The structure of the light source was symmetrical, so both ends of the electrode could be arbitrarily connected to the positive or negative poles of the power supply. Since silicon has the disadvantage of an indirect band gap, its radiation recombination efficiency is low. Therefore, carrier injection technology was adopted, and we used a mask plate to inject boron (with an injection energy of 60 keV) and phosphorus (with an injection energy of 100 keV) into the designated area, followed by annealing at 950 °C for 36 min to obtain P-type silicon and N-type silicon. The concentration of the P zone was 1.85 × 10^17^ cm^−3^, and the concentration of the N^+^ zone was 1.58 × 10^20^ cm^−3^. The polysilicon layer had four PN junctions in series, including two forward bias junctions and two reverse bias junctions, to improve the luminescence intensity. The doping concentration in the P region was much lower than that in the N^+^ region, where the electric field increased as the depletion region extended into the next N^+^ region. This configuration enhanced the avalanche multiplier effect in the low doping P region. The number of carriers decreased sharply in the first reverse bias junction due to the radiation recombination. This concentration increased in the following forward bias junction, and the recombination occurred again in the subsequent reverse bias junction.

**Figure 3:**
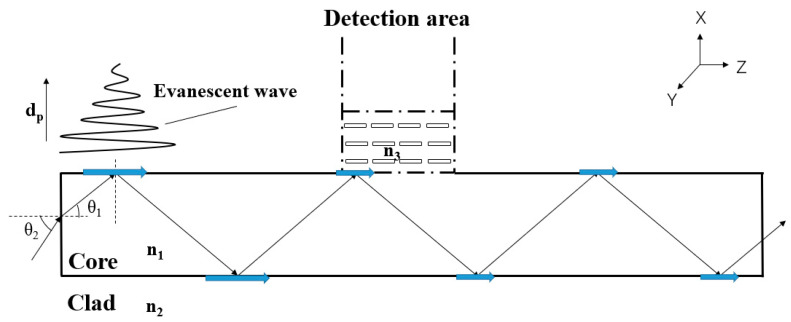
The light path in the OW.

Figure 3 presents the propagation path of light in the OW structure, which enters the core layer at an incident angle *θ*_2_ and a refraction angle *θ*_1_. After entering the OW, the light experiences total reflection in the core layer, according to Snell’s Law (1):

(1) $$\frac{n_{1}}{n_{2}}=\frac{\sin\theta_{2}}{\sin\theta_{1}}$$

In order to allow light to propagate in the OW with total reflection, the maximum *θ*_1_ is 43.82°, and the maximum *θ*_2_ is 73.67°.

When light is transmitted with total reflection in the core layer, the incident point and the reflection point are not at the same position, but are separated by several wavelengths, called the Goose–Hansen displacement [30]. This light wave, which flows along the cladding surface, is called an evanescent wave [31].

The amplitude of an evanescent wave is perpendicular to the interface direction and exhibits an exponential attenuation trend, which can be expressed as:

(2) $$E=E_{0}\exp\left(-x/d_{p}\right)$$

where *E*_0_ is the amplitude of the incident field, *x* is the distance between the light wave and the interface, and *d_p_* is the transmission depth, which is determined by Formula (3):

(3) $$d_{p}=\frac{1}{k_{0}\sqrt{n_{1}{}^{2}\;sin^{2}\theta -n_{2}{}^{2}}}$$

where *k*_0_ is the wave number, and *θ* is the incident angle.

The intensity of the evanescent wave decreases exponentially with the increase in the distance from the interface, so the evanescent wave exists only on the reflected surface. The scattering caused by the mode mismatch between the OW and the detecting material leads to light attenuation. In the detection area, the refractive index of the material to be detected should be at least greater than the clad layer.

**Figure 4:**
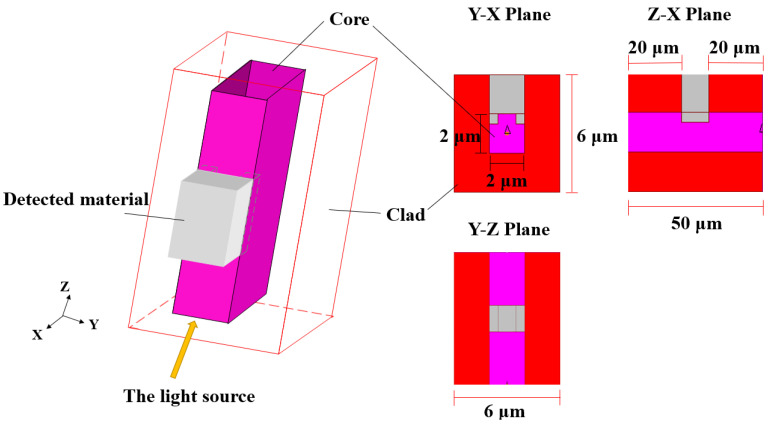
The structure of the embedded OW.

The OW structure transmission efficiency is crucial to achieving highly efficient light transmission. Since Si_3_N_4_ is a composite material with good radiation and heat stability, it shows great potential in addressing the limitations of silicon-based OWs, such as optical modulation and highly sensitive and low-loss sensors, and it is compatible with the CMOS process. In addition, Si_3_N_4_ is not affected by near-infrared nonlinear loss, because of its large bandwidth and high thermal damage threshold. In this paper, silicon dioxide and Si_3_N_4_ were combined to explore a new OW transmission structure. On the one hand, silicon dioxide acts as a cladding to confine light in the core layer. It also acts as an insulating layer between the Si_3_N_4_ and the silicon substrate. In addition, we used a strip-based OW structure in this paper, which is more suitable for the geometry of the semiconductor platform, to reduce the process difficulty. The OW structure is shown in Figure 4, with silicon dioxide as the cladding and Si_3_N_4_ as the core. The detection area was located in the middle of the OW. The large size of the core layer facilitated the light to enter the OW more easily, but the sensitivity of an ASPB increases with the reduction of the cross-sectional area of the OW [32]. Therefore, the size of the cross-sectional area required a proper trade-off. The OW structure comprised two parts. Si_3_N_4_ was covered between the material to be detected and silicon dioxide in the detection area, and the rest of the area was fully covered with silicon dioxide. It is worth noting that the detection structure designed in this paper was embedded in the core of the OW, which promoted the interaction between light and the material to be detected. Moreover, this kind of OW is called an embedded OW.

In addition, the detector structure used in this work was silicon-based. This type of detector has a mature process and can be well compatible with the light source and the OW.

## 3. Results and Discussion

**Figure 5:**
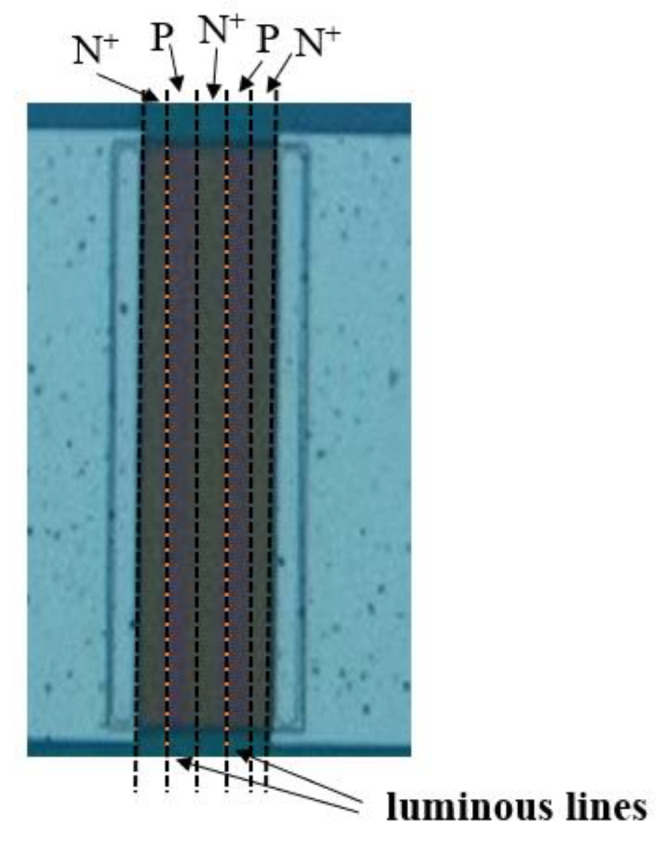
Micrograph of the light source under experimental conditions.

**Figure 6:**
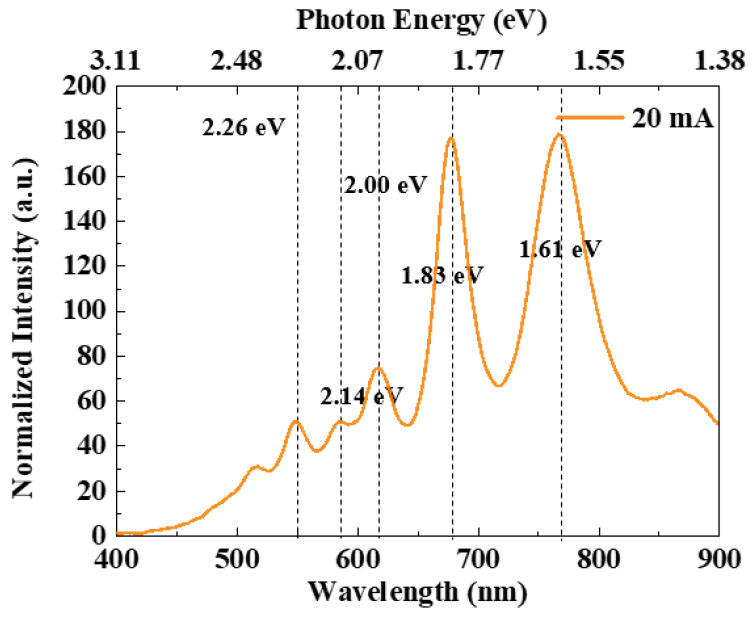
Electroluminescence spectra of the light source.

Figure 5 depicts the luminous lines. Figure 6 illustrates the electroluminescence spectra of the polycrystalline silicon cascade light source under a 20 V driving voltage and 20 mA driving current, which provided the conditions to simulate the OW propagation of this paper. In this condition, the light in the 400–900 nm wavelength range is stronger. Figure 6 highlights the presence of five peaks at wavelengths of 550 nm, 580 nm, 620 nm, 680 nm, and 770 nm. According to our analysis, the peak values at 2.26 eV and 2.14 eV may be due to direct band-to-band transitions between high-field carrier clusters. Defects introduced by carrier injection technology during the preparation phase could be the source of the 2 eV peak. The peak at 1.83 eV may be related to the indirect radiation recombination between the conduction band and the valence band, and the peak at 1.61 eV may be related to the transmission spectrum of the polysilicon layer [33].

Quantum efficiency is the ratio of the number of photons to the number of electrons emitted, directly affecting the light source’s brightness. The quantum efficiency *η_Q_* is calculated as follows:

(4) $$\eta_{Q}=\frac{P_{opt}/\left(h\upsilon \right)}{I/e}$$

where *P_opt_* is the optical power, *h* is the Planck constant, *υ* is the photon frequency, and *I* is the driving current. Under the experimental conditions, the total luminous power was 2.154 μW, and the quantum efficiency *η_Q_* = 5.78 × 10^−5^.

In addition, photoelectric conversion efficiency represents energy utilization. When the driver voltage is constant, the photoelectric conversion efficiency of the light source is proportional to the quantum efficiency. The calculation formula for photoelectric conversion efficiency is:

(5) $$\eta_{w}=\frac{P_{opt}}{IU}$$

where *U* is the driving voltage. The photoelectric conversion efficiency was 5.4 × 10^−6^. Table 1 compares the numerical results of various silicon-based light sources, revealing that the cascade light source had higher *η_Q_* and *η_w_*. 

In order to simplify the model, the light source with a wavelength of 680 nm was used in the simulation of the waveguide, and it was incident at an angle of 30° into the core layer of the OW. The simulation results demonstrated that the energy efficiency of the embedded OW can reach 95.7% (the loss was 0.19 dB). Under this condition, the refractive index of Si_3_N_4_ and silicon dioxide was n_1_ = 2.018 and n_2_ = 1.456. The relative refractive index difference between the cladding and the core was 23.97%, and a small refractive index difference could significantly reduce the multimode dispersion. Although some light energy escaped from the core layer in the form of evanescent waves, the majority of light energy was transmitted through the core layer.

Transmission loss (TL) is a highlight of this work, calculated by:

(6) $$\mathrm{TL}=10\;\mathrm{lg}\frac{P_{in}}{P_{out}}\;(dB)$$

where *P_in_* is the power of the incident light wave in OW, and *P_out_* is the power at the end of OW.

This paper used the finite-difference time-domain beam propagation method (FD-BPM) of the BeamPROW module during the simulations. The OW length was 50 μm, and the detection region was between 20 and 30 μm (Figure 4).

**Figure 7:**
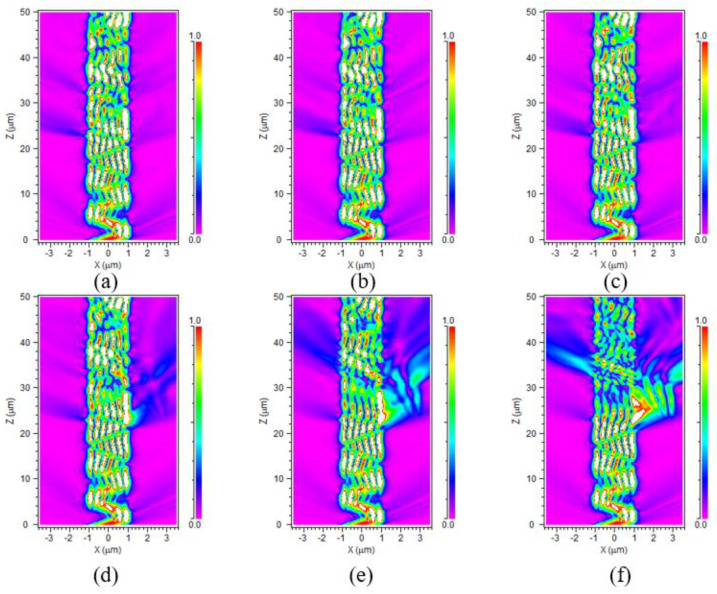
The light distribution of the OW when (**a**) n = 1.33, (**b**) n = 1.43, (**c**) n = 1.53, (**d**) n = 1.63, (**e**) n = 1.73, and (**f**) n = 1.83. n is the refractive index of the detected material.

In order to verify the feasibility of the embedded OW model, this work simulated the change in the light energy at the end of the OW through varying refractive indexes of the materials, which ranged from 1.33 to 1.83. Figure 7a–f illustrates the energy distribution in the core of the embedded OW when the detected materials had refractive indexes, respectively, of 1.33, 1.43, 1.53, 1.63, 1.73, and 1.83. In the X-Z plane, on the side where the detecting material was located (i.e., on the right side of the core layer), energy loss increased with the increasing refractive index when the refractive index of the detected material was greater than that of the cladding. However, it is worth noting that there was a clear correlation between energy dissipation and refractive index variation on the left side of the waveguide core layer when the refractive index of the detected material ranged from 1.63 to 1.83. This may be because the refractive index variation in the detection region caused a slight change in the angle of light propagation in the core layer, leading to the escape of energy from the left side of the core layer, and thus having higher sensitivity. Typically, loss increases with an increasing refractive index.

**Figure 8:**
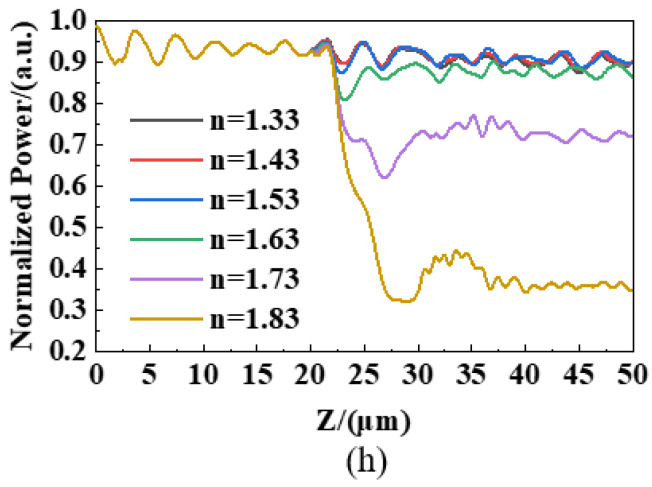
The dependence of the normalized output power of the embedded OW on refractive indexes of the detected material.

In Figure 8, the attenuation amplitude has almost no difference within the refractive index range of 1.33–1.53. For one thing, the refractive index of the detected material was smaller than that of the clad layer, so it was more difficult for light to spill out to the detected material. For another, it may be due to light scattering. The attenuation trend is obvious in the refractive index range of 1.63–1.83. Moreover, as the refractive index increased, the light energy loss was greater after a length of 20 μm, and the energy of the core layer decreased significantly. Furthermore, the core energy decreased faster as the refractive index increased.

**Figure 9:**
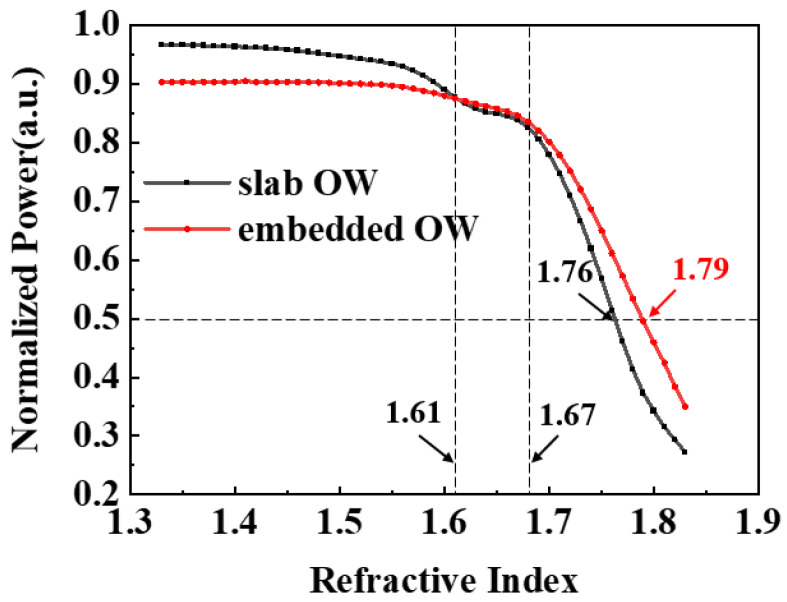
The comparison with the transmission loss of the embedded OW and that of the slab OW.

Compared to the conventional slab structure, the embedded OW structure designed in this paper had a lower detection loss. Specifically, we analyzed the energy distribution at the end of the waveguide for two types of structures with different refractive index materials and took the refractive index change gradient as 0.01. The normalized power of the two structures is depicted in Figure 9. The energy attenuation is obvious when it ranges between 1.61 and 1.67, and the light energy decreases from 87.5% to 84.5% in the embedded OW. This is because some light was transmitted into the detected material and dissipated. When the refractive index ranged from 1.68 to 1.83, the OW core energy decreased rapidly, and the light power decreased from 83.5% to 34.3%. When the refractive index exceeded 1.83, the energy loss was excessive, and it was difficult for the photodetector to identify the optical signal accurately. At the same time, the transmission loss of the embedded OW was smaller than that of the strip OW when the refractive index was greater than 1.61. For subsequent photodetectors, the lower the transmission loss in the OW, the more accurate the identification of optical signals. This work calculated that when the refractive index range was 1.61–1.67, the power decreased by 1.1%/0.01 RIU. When the refractive index range was 1.68–1.83, the energy decreased by 3.29%/0.01 RIU.

**Figure 10:**
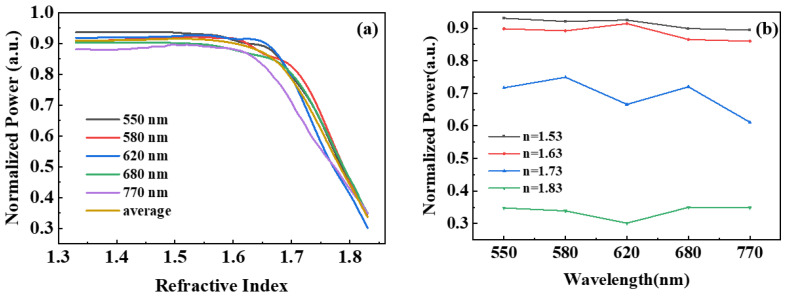
The dependence of the normalized output power of the OW on (**a**) the refractive index of detected material and (**b**) the wavelength of light.

To confirm the accuracy of the ASPB, we simulated the light of the four other peak wavelengths of the PN junction cascade light source spectrum as the incident light source under the same experimental conditions. The relationship between the power at the end of the waveguide and the refractive index is illustrated in Figure 10a, revealing that the variation trend of these curves is consistent with those of a 680 nm wavelength light source. Further details are reported in Table 2, highlighting that the detection characteristics of the ASPB are consistent with the rules presented in the previous analysis. In addition, for the optical transmission characteristics of the ASPB, the average power value of all peaks in the light source spectrum represents the performance of the ASPB. When the refractive index of the detected material exceeded 1.52, the energy decreased with the increase of the refractive index, showing monotonicity. Figure 10b infers that when the refractive index increases, the energy attenuation trend increases, and the sensitivities of different ranges were calculated as shown in Table 3. The simulation results highlighted that the ASPB could detect some organic solvents in the laboratory, such as chlorobenzene, nitrobenzene, and bromobenzene. A detailed comparison of the ASPB with other types of sensors is presented in Table 4. Although the sensitivity of the ASPB was not outstanding, its monolithic integrated characteristic is worth further investigation.

## 4. Conclusions

In this paper, we designed an ASPB light source and OW, which affords a luminous efficiency of 5.4 × 10^−6^ and a quantum efficiency of *η_Q_* = 5.78 × 10^−5^ under normal working conditions. Based on the CMOS process platform, the designed light source and OW can achieve all-silicon monolithic integration. When the refractive index of the detected material exceeds 1.61, the detection loss of the embedded OW is smaller than that of the slab OW. In addition, by simulating the five peak wavelengths of light sources in the spectrogram, the changes in light energy caused by materials with different refractive indexes were detected, suggesting that the fitted average power could represent the performance of the ASPB, and materials with a refractive index greater than 1.52 could be detected. Due to the low loss, high detection accuracy, low cost, and fast speed, the ASPB based on the PN junction cascade light source and the embedded OW can be applied in many fields, such as detecting different organic solvents in the lab.

## Figures and Tables

**Table 1:** Comparison of properties of several silicon-based light sources.

Type	*η_Q_*	*η_W_*	Driving Voltage	Driving Current	References
MOS_like	1.47 × 10^−7^	8.03 × 10^−9^	42 V	55 mA	[34]
Si_LED	\	2.4 × 10^−8^	9.8 V	130 mA	[35]
Cascade	5.78 × 10^−5^	5.4 × 10^−6^	20 V	20 mA	This work

**Table 2:** The impact of light wavelength on the detection range.

Wavelength	Si_3_N_4_	SiO_2_	Detection Range	Refractive Index
	n_1_(Core)	n_2_(Clad)		(TL = −3 dB)
550 nm	2.033	1.46	n > 1.47	n = 1.78
580 nm	2.029	1.459	n > 1.53	n = 1.79
620 nm	2.024	1.457	n > 1.55	n = 1.77
680 nm	2.018	1.456	n > 1.46	n = 1.79
770 nm	2.012	1.454	n > 1.52	n = 1.77
Average	\	\	n > 1.52	n = 1.78

**Table 3:** The dependence of our device’s sensitivity on the refractive index of the material to be detected.

Wavelength	Sensitivity		
	n < 1.63	1.63 < n < 1.73	1.73 < n < 1.83
550 nm	1.14 dB/RIU	9.80 dB/RIU	31.39 dB/RIU
580 nm	1.38 dB/RIU	7.57 dB/RIU	34.47 dB/RIU
620 nm	0.71 dB/RIU	13.73 dB/RIU	34.48 dB/RIU
680 nm	2.62 dB/RIU	7.98 dB/RIU	31.35 dB/RIU
770 nm	1.57 dB/RIU	14.82 dB/RIU	24.42 dB/RIU
Average	1.26 dB/RIU	10.68 dB/RIU	31.26 dB/RIU

**Table 4:** Comparison of the ASPB with the reported sensors with different structures.

Sensing Structure	Maximum Sensitivities	References
Fused fiber taper	6.41 dB/RIU	[36]
Fabry–Perot interferometer	52.4 dB/RIU	[37]
Michelson fiber	30.11 dB/RIU	[38]
ASPB	31.26 dB/RIU	This work
